# Grate-Fired Biomass Combustion Plants Using Forest Residues as Fuel: Enrichment Factors for Components in the Fly Ash

**DOI:** 10.1007/s12649-016-9565-6

**Published:** 2016-04-27

**Authors:** Christof Lanzerstorfer

**Affiliations:** School of Engineering/Environmental Sciences, University of Applied Sciences Upper Austria, Stelzhamerstraße 23, 4600 Wels, Austria

**Keywords:** Biomass ash, Grate-fired combustion, Nutrients, Heavy metals, Enrichment factor

## Abstract

The aim of this work is to investigate the enrichment factors for various nutrients and heavy metals in the fly ash from grate-fired combustion plants using forest residues as fuels. Sustainable energy production requires recycling of the ash on the soil to close the nutrient cycles. The coarser bottom ash which is discharged from the boiler usually contains lower amounts of heavy metals compared to the fly ash which is separated from the off-gas. The discharge of the finest fly ash to landfill sites serves to remove the unwanted heavy metals from the cycle. For this purpose, the enrichment of these components in the finest fly ash should be maximized. At the same time the enrichment of the nutrients in the fine fly ash should be minimal. The enrichment of the components in the electrostatic precipitator fly ash of three biomass combustion plants was determined. The enrichment of the critical heavy metals Cd, Pb and Zn in the fly ash was higher than the values reported in one study but less than values calculated from the data presented in another study. Further investigations would be required to clarify this deviation. Thereby additional data on the furnace operation conditions e.g. combustion temperature should be included. The enrichment of most nutrients (Ca, Mg and PO_4_^3−^) in the fly ash was low. For K the enrichment in the fly ash was higher resulting in notable K losses to the fly ash.

## Introduction

The use of forest residues as fuel for the generation of heat and power in biomass combustion plants is continuously becoming more widespread [1]. The increase in the use of forest residues for energy production is causing a growing demand for fuel. However, the harvesting of biomass material leads to a loss of nutrients like Ca, Mg, K and P in the soil which requires compensation. The application of fertilizers is quite costly. Therefore, the process of recycling the ash produced during combustion back into the earth is investigated to close the nutrient cycles for the soil. Thus, sustainable biomass fuel utilization can be realized [2, 3].

The ash from biomass combustion also contains heavy metals. In several countries the concentration of various heavy metals in biomass ash recycled into the soil is limited by regulations. The components which are usually critical with respect to these regulations are As, Cd, Pb and Zn [4–6]. In Austria the application of electrostatic precipitator (ESP) fly ash or filter fly ash is even prohibited [7] because they contain the highest concentrations of heavy metals [8]. This fly ash is usually landfilled. The reason for the enrichment of these heavy metals in the fly ash, especially in the finest fractions, is that they are volatilized to some extent at combustion temperature and re-condensed on the fly ash when the off-gas cools down. Model calculations for the chemical equilibrium of various nutrients and heavy metals under biomass combustion conditions have been reported [9]. These calculations suggest that Cd, Cu and Pb should be efficiently volatilized during combustion and to a lesser extent As, Cr and Zn, whereas Ni and V are not volatilized. A high enrichment of heavy metals in the ESP fly ash in combination with a low amount of fly ash would be advantageous to remove the heavy metals from the cycle efficiently.

In literature only limited information is available on measured results of the enrichment of heavy metals in the fly ash from grate-fired forest residue combustion. The enrichment can be quantified by the enrichment factor (EF) which is defined by the concentration of a component in the fly ash divided by the concentration of this component in the bottom ash. A value of the EF of >1 indicates that the component is enriched in the fly ash, while for a component depleted in the fly ash the value is <1. Reported EFs for inorganic compounds in the cyclone fly ash from a 6 MW combustion plant using forest residues as fuel are shown in Table 1 [10]. EFs can also be calculated from the reported concentration data. In another study average concentration data for some heavy metals in bottom ash, cyclone fly ash and filter fly ash from the grate-fired combustion of bark, wood chips and sawdust are summarized [11]. For some elements the calculated EFs for cyclone fly ash are in agreement with the EFs reported [10]. For other elements, however, there is a considerable deviation. For filter fly ash collected in a second stage dust separator downstream of the cyclone and therefore consisting of much smaller particles, the calculated EFs are considerably higher for the volatile components Cd, Pb and Zn (Table 1).

**Table 1:** Published enrichment factors for various components

	[10]	[11]	
	Reported EF cyclone fly ash/bottom ash	Calculated EF cyclone fly ash/bottom ash	Calculated EF filter fly ash/bottom ash
P	3.1	–	–
S	10.5	–	–
Ca	1.6	–	–
Mg	1.5	–	–
Na	1.4	–	–
K	2.9	–	–
Fe	0.8	–	–
Mn	1.3	–	–
As	0.3	–	–
Ba	1.9	–	–
Cd	4.4	18	67
Co	1.2	–	–
Cr	0.9	0.5	0.7
Cu	1.0	0.9	2.4
Ni	1.0	0.9	1.0
Pb	2.6	4.2	77
Ti	0.2	–	–
V	0.9	–	–
Zn	3.8	4.3	30

The exclusion of the fly ash from recycling on the soil serves to deplete in the soil those heavy metals which are enriched in the fly ash. A maximized enrichment of the heavy metals in the fly ash is required when the biomass used as fuel in the combustion plant has grown on contaminated soil. This biomass as well as the ash produced in the combustion process is likely to be more highly contaminated with heavy metals [12, 13].

The aim of this study was to obtain more information on the enrichment of components in the filter fly ash from grate-fired combustion plants using forest residues as fuel. For the calculation of EFs ash samples from three similar combustion plants were analyzed. These plants consist of a grate-fired combustion unit and a boiler followed by a two stage off-gas cleaning system with a multi-cyclone as the first stage and an ESP as the second stage. The fly ash collected in the multi-cyclone is discharged together with the bottom ash as mixed ash, whereas the ESP fly ash is discharged separately.

## Materials and Methods

### Ash Samples

The mixed bottom and cyclone fly ash samples were collected from the dry ash discharge of the boiler. The ESP fly ash samples were taken from the outlet of the fly ash conveyor of the ESP. Approximately 2 dm^3^ of each of the ash samples was collected. An overview of the thermal capacities of the plants as well as the combustion temperature and the combusted biomass is given in Table 2. The volume of the ash samples was reduced to a volume suitable for the various laboratory tests using sample dividers which were applied repeatedly (Haver&Boecker HAVER RT, Quantachrome Micro Riffler).

**Table 2:** Biomass combustion ashes investigated

	Thermal capacity (MW)	Combustion temperature (°C)	Combusted biomass
Plant A	10	830	Wood chips from forest residue. 80 % softwood
Plant B	10	900	Wood chips from forest residue. 85 % softwood
Plant C	10	920	Wood chips from forest residue. 90 % softwood

In fly ashes the concentration of various components has a high correlation with the particle size [14, 15]. The particle size of the ESP or filter fly ashes depends very much on the separation efficiency of the cyclone pre-separator. Thus, the enrichment of these components in the ESP fly ash could also be influenced by the pre-separator. Therefore, the particle size distribution of the ESP fly ashes was measured to identify any differences between the dust separation characteristics at the various plants.

### Chemical Analysis

The particle size distribution of the fly ash samples was measured using a laser diffraction instrument with dry sample dispersion from Sympatec, type HELOS/RODOS. The particle size distribution of the mixed bottom ash and cyclone ash samples was determined using the laboratory sieve shaker with sieves from 4 mm to 500 µm. The undersize material (<500 µm) was analyzed using the laser diffraction instrument.

The moisture content of the ash samples was determined gravimetrically. The samples were dried at 105 °C for 1 h. All chemical analyses were determined by testing each sample in duplicate. In the results the average values are presented. Prior to analysis the mixed bottom ash and cyclone ash samples were sieved at 2.5 mm to remove the oversize fraction and subsequently milled in a Retsch mixer mill MM301 with agate grinding tools.

To determine the concentration of metals, sulfate and phosphate in the fly ash the solid samples were dissolved by aqua regia digestion prior to analysis. The concentration of Na, K, Mg, Ca, SO_4_^2−^ and PO_4_^3−^ was measured by ion chromatography. The metals were measured by inductively coupled plasma optical emission spectroscopy (ICP-OES). For the analysis an ICP-OES system Ultima 2 from Horiba Jobin–Yvon was used. The analytical methods are described in detail elsewhere [6].

Chloride cannot be analyzed after digestion by aqua regia. However, the chlorides of all metals present in the fly ash at relevant concentrations are highly soluble in water. Therefore the concentration of chloride was determined by leaching the samples in water and subsequent measurement by ion chromatography. The maximum chloride concentration in the aqueous leachate was approximately 200 mg/dm^3^, which is very low compared to the solubility of the chlorides.

## Results and Discussion

### Particle Size Distribution

**Fig. 1:**
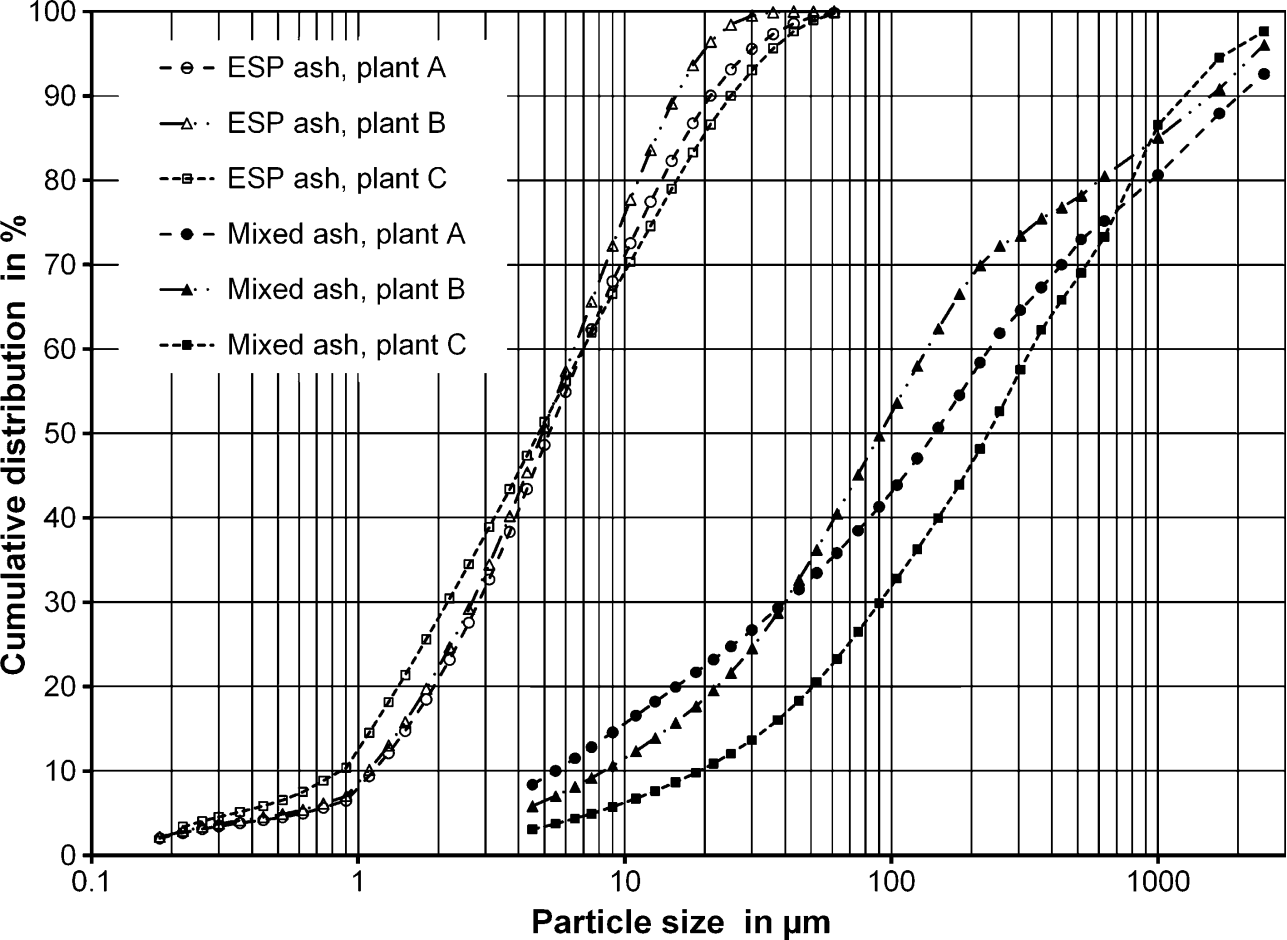
Particle size distribution of the ESP fly ashes and the mixed bottom and cyclone fly ash samples

The particle size distribution of the ash samples are shown in Fig. 1. The size distributions of the three ESP ashes are very similar and the mass median diameters are all close to 5 µm. Therefore, no major influence of the separation efficiency of the cyclone pre-separator on the EFs has to be expected. The mixed bottom and cyclone fly ashes are much coarser and their particle size distributions differ more. The mass median diameters of the mixed ashes are in the range of 90–220 µm.

### Chemical Composition

The results of the chemical analysis of the ashes are summarized in Table 3. The concentrations of most components were very similar in the ashes from the various plants. However, the concentrations of Na, Cl^−^, Cd, Pb and Zn were considerably higher in the ashes from plant C.

**Table 3:** Chemical composition of the ashes (all concentrations based on dry weight)

		Mixed bottom and cyclone fly ash			ESP fly ash		
		A	B	C	A	B	C
Cl^−^	g/kg	0.44	0.80	5.2	28.0	47.2	198
PO_4_^3−^	g/kg	22.7	14.8	22.7	46.3	41.5	23.5
SO_4_^2−^	g/kg	9.1	9.0	11.0	155	96.0	94.2
Ca	g/kg	132	125	80.1	161	228	104
Mg	g/kg	14.1	18.9	7.5	24.0	28.4	10.7
Na	g/kg	2.2	2.0	7.8	2.7	2.3	39.4
K	g/kg	41.7	29.1	25.2	138	81.2	143
Al	g/kg	19.6	22.5	19.4	7.8	8.7	8.4
Fe	g/kg	12.0	15.8	13.2	8.3	8.5	11.2
Mn	g/kg	8.5	3.4	4.8	13.8	7.7	7.2
As	mg/kg	47	54	49	48	57	81
B	mg/kg	136	131	133	662	623	371
Ba	mg/kg	1020	617	911	206	316	189
Cd	mg/kg	2	1	6	40	31	75
Co	mg/kg	7	4	2	12	8	4
Cr	mg/kg	44	50	39	72	102	58
Cu	mg/kg	57	69	107	145	177	207
Mo	mg/kg	5	5	4	13	8	6
Ni	mg/kg	31	39	17	46	49	28
Pb	mg/kg	15	17	52	259	307	911
Sb	mg/kg	10	9	8	9	7	20
Sr	mg/kg	494	334	277	653	734	396
Ti	mg/kg	974	1150	1040	400	457	542
V	mg/kg	29	37	19	21	33	16
Zn	mg/kg	275	292	992	7270	4310	14,800

### Enrichment Factors

The calculated EFs are summarized in Table 4 together with the mean and the standard deviation. For some components the EFs are less than 1. That means that these components are depleted in the ESP fly ash. This is true for Al, Fe, Ba Ti and V. For many components the EFs are in the range of 1.0–2.0 which indicates a slight enrichment of these components in the ESP fly ash. Considering the standard deviation of the EFs there might even be no enrichment at all. Such components are PO_4_^3−^, Ca, Mg, Na, Mn, As, Co, Cr, Mo, Ni, Sb and Sr. Components with a distinct enrichment (EF from 2.0 to 10) were K, B, and Cu. A significant enrichment with EFs higher than 10 was found for Cl^−^, SO_4_^2−^, Cd, Pb and Zn.

**Table 4:** Enrichment factors

	EF			EF mean	EF standard deviation
	A	B	C		
Cl^−^	63	59	38	52	13
PO_4_^3−^	2.0	2.8	1.0	2.0	0.9
SO_4_^2−^	17	11	8.6	12	4.4
Ca	1.2	1.8	1.3	1.4	0.3
Mg	1.7	1.5	1.4	1.5	0.1
Na	1.2	1.1	5.0	2.5	2.2
K	3.3	2.8	5.7	3.9	1.5
Al	0.40	0.38	0.43	0.41	0.02
Fe	0.69	0.54	0.84	0.69	0.15
Mn	1.6	2.3	1.5	1.8	0.4
As	1.0	1.0	1.7	1.2	0.4
B	4.9	4.7	2.8	4.1	1.2
Ba	0.20	0.51	0.21	0.31	0.18
Cd	20	23	13	18	5.2
Co	1.7	1.9	2.3	2.0	0.3
Cr	1.6	2.1	1.5	1.7	0.3
Cu	2.5	2.6	1.9	2.3	0.4
Mo	2.7	1.6	1.5	1.9	0.6
Ni	1.5	1.3	1.6	1.1	0.2
Pb	17	18	18	18	0.6
Sb	0.89	0.78	2.4	1.4	0.9
Sr	1.3	2.2	1.4	1.6	0.5
Ti	0.4	0.4	0.5	0.44	0.1
V	0.73	0.90	0.84	0.82	0.08
Zn	26	15	15	19	6.7

The relative standard deviation for the EFs was less than 30 % for most of the components (Ca, Mg, Al, Fe, Mn, As, B, Cd, Co, Cr, Cu, Ni, Pb, Sr, Ti and V). For Cl^−^, SO_4_^2−^, PO_4_^3−^, K, Mo and Zn the relative standard deviation was between 30 and 50 %. The highest values for the relative standard deviation (>50 %) for the EF were found for Na, Ba and Sb.

The EFs for the main components Ca, Mg, K, Fe, Mn and SO_4_^2−^ are quite similar to the EFs reported [10] although these EFs were measured for cyclone fly ash. For Na and PO_4_^3−^ the EFs for plant A and B are also similar to those reported [10], but for plant C the EFs deviate considerably. Also the EFs for V and Ti correspond.

For As a slight enrichment was found in the ESP fly ash, whereas in [10] a depletion of As in the cyclone fly ash was reported. For Ba the results are vice versa. In this study a considerable depletion was measured while in [10] an increased concentration was found in the fly ash.

For Co the EF found in this study was somewhat higher. For Cr and Ni a slight enrichment was found for the fly ash, whereas in both studies [10, 11] a slight depletion of these components in the fly ash was reported.

The EF for Cu in this study was similar to that calculated from the data reported in [11] for filter fly ash. As there was no enrichment of Cu reported for the cyclone ash, the difference in the calculation basis is irrelevant.

For the critical components Cd, Pb and Zn the measured EFs are much higher than the EFs reported for cyclone fly ash [9]. On the other hand, the values are much lower than those calculated from the data reported for filter fly ash [11]. This can be explained by the different calculation basis for the EFs. In this study the concentration of the ESP fly ash is related to the concentration in the mixed bottom ash and cyclone fly ash, whereas the EFs calculated from the data reported in [11] are related to the concentration in the bottom ash. As these components are enriched already in the cyclone fly ash, the concentration in the mixed bottom ash and cyclone fly ash used in this study is increased and, therefore, the EFs are lower. Other reasons for the deviations in the enrichment factors could be differences in the combustion temperature, in the off-gas oxygen content or in the ratio of fly ash produced to bottom ash. However, these data are not available in the published studies.

## Conclusions

For sustainable wood-based energy production recycling of the ash on the soil is important to close the nutrient cycles. The enrichment of unwanted heavy metals in the fine fly ash should be maximized, while no enrichment should incur for the nutrients. The enrichment of the critical components Cd, Pb and Zn in the filter fly ash was high, whereas for other heavy metals (As, Co, Cr, Cu, Mo and Ni) the enrichment was quite low. The enrichment factors for Cd, Pb and Zn were higher than the values reported in one study but less than values calculated from the data presented in another study. Therefore, further investigations are required which should also include more detailed data on the furnace operation conditions like the combustion temperature, the off-gas oxygen content and the moisture content for optimization of the enrichment of heavy metals in the fine fly ash. The enrichment of most nutrients (Ca, Mg and PO_4_^3−^) in the fly ash was found to be low. Solely for K was the enrichment factor higher resulting in notable K losses to the fly ash.
